# Work economic sectors and cardiovascular risk factors: cross-sectional analysis based on the RECORD Study

**DOI:** 10.1186/1471-2458-14-750

**Published:** 2014-07-24

**Authors:** Antoine Lewin, Frédérique Thomas, Bruno Pannier, Basile Chaix

**Affiliations:** Sorbonne Universités, UPMC Univ Paris 06, UMR_S 113, Institut Pierre Louis d’Epidémiologie et de Santé Publique, Paris, 75012 France; Inserm, UMR_S 1136, Institut Pierre Louis d’Epidémiologie et de Santé Publique, Paris, 75012 France; Centre d’Investigations Préventives et Cliniques, 6 rue La Pérouse, Paris, 75116 France

**Keywords:** Cardiovascular risk factors, Socioeconomic factors, Work economic sectors

## Abstract

**Background:**

Little is known on the comparative effect of work economic sectors on multiple cardiovascular risk factors. Such information may be useful to target Public health interventions, e.g., through the occupational medicine. We investigated whether and how a large panel of cardiovascular risk factors varied between 11 work economic sectors.

**Methods:**

Data on 4360 participants from the French RECORD Study geolocated at their residence were analyzed. Ten outcomes were assessed: body mass index (BMI), waist circumference, systolic and diastolic blood pressure (BP), pulse pressure, total cholesterol, glycaemia, high-density lipoprotein (HDL) cholesterol, low-density lipoprotein (LDL) cholesterol, and resting heart rate. Multilevel linear regression models stratified by sex and adjusted for individual and neighborhood sociodemographic characteristics were estimated.

**Results:**

Among men, the Health and social work sector was found to be the most protective sector for BMI, waist circumference, and glycaemia (while the Construction sector and the Transport and communications sector tended to be unfavorable for these outcomes). The Health and social work sector was also associated with higher HDL cholesterol among men. However, men working in the Health and social work sector showed the highest systolic BP and pulse pressure. Women working in the Health and social work sector had the highest BMI, the largest waist circumference, and the most elevated systolic and diastolic BP. The Commercial and repair of vehicles sector, the Transport and communication sector, and the Collective, social, and personal services sector were associated with a more favorable profile for these risk factors among women.

**Conclusion:**

Work economic sectors contribute to shape metabolic and cardiovascular parameters after adjustment for individual/neighborhood sociodemographic characteristics. However, patterns of associations varied strikingly according to the risk factor examined and between men and women. Such findings may be useful to target interventions for reducing cardiovascular risk, e.g., through the occupational medicine.

**Electronic supplementary material:**

The online version of this article (doi:10.1186/1471-2458-14-750) contains supplementary material, which is available to authorized users.

## Background

In Europe and North America, low socioeconomic status groups have a higher prevalence of chronic disease risk factors such as smoking, physical inactivity, obesity, hypertension, hypercholesterolemia, and diabetes [1–3], and a higher cardiovascular risk score [2, 4].

Associations are well established between various categorizations of occupation and related social class and cardiovascular risk factors [5–9]. Based on occupational classifications that distinguish blue-collar from white-collar workers or professional, technical, and manual workers [5, 9], there is substantial evidence for an inverse relationship between the occupational social level and cardiovascular disease risk factors [10] such as high blood pressure [5, 9, 11, 12], smoking [13–16], cholesterol [17], hemostatic factors [18], and obesity [19].

Compared to occupational social class, very few studies have focused on work economic sectors. Work economic sectors, even considered on a broad scale, may be of importance, because numerous exposures may vary in prevalence from one sector to the other. Studies have suggested that occupational exposures such as psychosocial factors [20, 21], work hardness, high physical demand, noise at work [21, 22], working rhythm [23, 24], and the prevalence of health behavior such as tobacco [25], alcohol, and drug consumption [26] show a different prevalence in the various work economic sectors [27].

The present work is grounded on the idea that, in addition to this flourishing literature investigating the relationship between specific occupational exposures and health; it is relevant to consider the broad economic sectors where people work. In Social epidemiology, it is very common to examine causes that are distant from the health outcomes investigated, with a distinction between the so-called upstream determinants (e.g., social class, work economic sectors) and downstream determinants of diseases that mediate the effects of the former. Considering work economic sectors in addition to social class and other broad characteristics of populations may be useful to identify an additional marker of populations in poor health, in order to target interventions to populations who critically need them. The operational interest of work economic sectors for targeting interventions is also related to the fact that this information is commonly reported and available in a number of databases. Finally, work economic sectors are also connected to a potential approach to develop interventions, through the occupational medicine departments represented in each company. Overall, documenting relationships between work economic sectors and cardiovascular risk factors is potentially important to develop Public health interventions.

Regarding cardiovascular health as the focus of the present article, one study investigated work economic sectors in relation to hypertension. Takashima et al. found that the age-adjusted prevalence of hypertension among transportation and communication workers was four times higher than that of service workers, who had the lowest prevalence of hypertension [11]. In another study, occupation-based social class and work economic sectors were combined into one classification, not allowing the authors to disentangle their independent effect on hypertension [7].

One study examined work economic sectors in relation to obesity in a Dutch working population [28]. The study found that there were three sectors with a relatively low body mass index (BMI): the catering industry, the healthcare sector, and the culture, sport, and recreation sector. On the opposite, workers of the transportation sector showed a high BMI and a high prevalence of overweight and obesity.

Only one study conducted among women examined work economic sectors in relation to multiple cardiovascular risk factors including cholesterol, blood pressure, heart rate, and anthropometric variables aggregated in order to reflect allostatic load [29]. The study found that working in the health care sector was associated with a higher allostatic load than working in the information technology and media sector. However, this study only compared two work economic sectors.

To our knowledge, no study investigated the association between a panel of different work economic sectors and multiple cardiovascular risks factors. Moreover, no study adjusted the relationships between work economic sectors and health for individual and neighborhood socioeconomic characteristics or compared the associations between men and women. Overall, our aim was to investigate whether and how a large panel of cardiovascular risk factors including weight status and fat, blood pressure (BP), cholesterol, glycaemia, and resting heart rate varied between 11 work economic sectors after adjustment for individual and neighborhood sociodemographic characteristics in a large working population.

## Methods

### Study population

#### The RECORD Study

Data from the first wave of the RECORD (Residential Environment and CORonary heart Disease) Cohort Study (http://www.record-study.org) were used for cross-sectional analyses. As described elsewhere [30–39], 7,290 participants aged 30–79 years were recruited without *a priori* sampling in 2007–2008 during free preventive medical checkups conducted by the Centre d’Investigations Préventives et Cliniques in the Paris metropolitan area. As an *a priori* eligibility criterion, only participants residing in 10 (out of 20) administrative districts of Paris or in 111 other municipalities in the region were selected for the study. Of the persons selected for participation, 83.6% agreed to participate and completed the data collection protocol.

#### Working participants with information on their workplace

Administrative files from CNAV (National Old Age Insurance System) were used to identify the establishment of work. We relied on a database of occupational careers routinely used for the computation of retirement pensions. The file received from CNAV indicated the employer (or employers, with a maximum of 3) of each participant for each year, with the corresponding establishment identification code and work economic sector. The file did not provide information on the dates of beginning and end of the contracts during the year. The data therefore did not allow us to confirm for sure that the participant was employed, nor with which employer he/she was employed (if several employers were reported), at the exact date of enrollment in the study. We retained for every year only the main employer, which was the one from which the participant received the most important salary. To be sure to only consider a workplace where the participant was already working (or had worked) at the time of recruitment in the study (and thus avoid reverse causality problems), we assigned to each individual the main work establishment of the year preceding his/her inclusion in the study.

After excluding participants with missing information on the work economic sector (n = 176), the final sample used for the analyses comprised 4,360 participants residing in the Ile-de-France region. Descriptive information on study participants is presented in Additional file 1. They were living in 648 census tract neighborhoods (defined by Insee by grouping three census block group neighborhoods) [40]. These census tracts comprised an average of 6.8 participants, and an average of 8205 residents. The study protocol was approved by the French Data Protection Authority.

### Measures

#### Cardiovascular risk factors

In order to assess a complete panel of the basic cardiovascular risk factors, ten outcomes were examined in this study [41]: body mass index (BMI), waist circumference, systolic and diastolic blood pressure (SBP and DBP), pulse pressure, total cholesterol, glycaemia, high-density lipoprotein (HDL) cholesterol, low-density lipoprotein (LDL) cholesterol, and resting heart rate. Height (using a wall-mounted stadiometer) and weight (using calibrated scales) recorded by a nurse during the medical examination were used to calculate BMI (kg/m^2^) [42, 43]. Waist circumference was measured in cm using an inelastic tape placed midway between the lower ribs and iliac crest on the midaxillary line [44]. Supine brachial BP was measured by trained nurses three times in the right arm after a 10 minute rest period, using a manual mercury sphygmomanometer [42]. SBP and DBP were defined as the first and fifth Korotkoff phases, respectively, using the mean of the last two BP measurements [44]. Pulse pressure was defined as the difference between systolic and diastolic BP. Total cholesterol, HDL cholesterol and glycaemia (enzymatic method, automat Hitachi 917, Hitachi, Tokyo, Japan) were measured under fasting conditions. LDL cholesterol was calculated from the Friedewald equation [45]. Trained nurses measured resting heart rate in bpm by electrocardiogram, using a Cardionics CardioPlug device. The measurement was made in a quiet room after a 5- to 7-mn rest period in the supine position [46].

#### Individual sociodemographic variables

Several sociodemographic characteristics were considered. The age of the participants was divided into 3 classes: 30–44, 45–59, and 60–79 years. Personal education was divided into four classes: no education, primary education and lower secondary education, higher secondary education and lower tertiary education, and upper tertiary education. Regarding occupation, four categories were distinguished: blue-collar workers, low-white collar workers, intermediate occupations, and high white-collar workers. Household income adjusted for household size was divided into four categories based on the quartiles. Marital status was coded in two classes (living alone or in couple). Self-reported financial strain (reporting financial difficulties) was coded as a binary variable.

#### Antihypertensive medication use

Antihypertensive medication use was determined by merging the administrative SNIIR-AM national health insurance database on all healthcare reimbursements in 2006–2009 to the RECORD Study database at the individual level. A binary variable was created indicating whether or not individuals had been reimbursed for any antihypertensive medication over the previous year.

#### Neighborhood socioeconomic variable

We hypothesized that neighborhood socioeconomic status may confound the relationship between work economic sectors and cardiovascular risk factors (if the place where people live influence individuals’ job opportunities). An alternative hypothesis may be that neighborhood socioeconomic status intervenes as a mediator in the relationship between work economic sectors and cardiovascular risk (if work economic sectors, through their influence on the salary, determine where people can afford to live). Under the latter hypothesis, neighborhood socioeconomic status may be seen as an “indirect biasing pathway” and would also have to be adjusted for, because the effect of work economic sectors we are interested to isolate operates independently of the socioeconomic status of the neighborhood [47, 48].

The socioeconomic status of the neighborhood was assessed with the educational level of the residents. The variable was computed within buffers with a radius of 1000 m centered on participants’ residences. These buffers took into account the street network, i.e., comprise the area that is accessible within 1000 m along the street network. ArcInfo10 and its Network analyst applied to street network data from the National Geographic Institute were used to derive such buffers.

#### Work economic sectors and legal category of the establishment

Based on the business identification code of each workplace retrieved from the CNAV data, we then used databases of facilities or companies from Insee (Permanent Database of Facilities, SIRENE register) or Trade Dimension to identify the work economic sector of each participant. We used the 17 levels of the 2003 French classification of activities (NAF) to classify work economic sectors. Of those 17 levels of activities, only 11 were represented in our sample: Health and social work; Manufacturing industry; Construction; Commercial, repair of motor vehicles and motorcycles; Hotels and restaurants; Transport and communications; Financial activity; Real estate, renting, and business services; Public administration; Education; and Collective, social and personal services. The Health and social work sector was taken as the reference category because preliminary descriptive analyses showed that this sector showed both the lowest level of risk for certain cardiovascular risk factors and the highest level of risk for other risk factors, thus that it was relevant to report the findings as contrasts with this particular sector.

It is important to note, first, that the work economic sector was assigned at the level of the establishment or worksite (the establishment is an economic entity located in a definite location and subordinated to a unique authority) rather than at the broader level of the company. Second, worksites were classified according to the main economic activity of the establishment. Accordingly, cleaning services or administrative services integrated in a company from the Construction sector would be classified as part of the Construction sector. Third, the work economic sector is independent of the personal social group of the person, and each work economic sector comprises people of different classes (e.g., managers and blue-collar workers, low and high white collar workers).

Additional analyses were conducted with the work economic sectors combined into a smaller number of groups: the primary sector (extraction and production of raw materials), the secondary sector (transformation of raw or intermediate materials into goods) and the tertiary sector (supply of services to consumers and businesses). The primary sector was excluded from the analyses because of a too small sample size (n = 14 for men and n = 14 for women).

The Insee databases also enabled us to distinguish between the private and the public sector.

### Statistical analysis

Multilevel linear regression models with a random effect at the census tract level were estimated to account for within-neighborhood correlation in the cardiovascular risk factors examined. Given differences in occupation and cardiovascular profiles between females and males, all the analyses were stratified by sex. Models of the relationships between work economic sectors and cardiovascular risk factors were adjusted for individual sociodemographic variables and neighborhood education level. Models for blood pressure were further adjusted for antihypertensive medication use. All the analyses were conducted with SAS 9.3 (SAS Institute, Cary, North Carolina).

## Results

The associations between individual or neighborhood sociodemographic variables and cardiovascular risk factors are shown in Additional files 2, 3, 4 and 5. The most consistent associations were documented with age, individual education, and neighborhood education. All risk factor variables increased with age, and HDL cholesterol also increased with age. In men and/or women, a low personal education was associated with a higher BMI, waist circumference, SBP, DPB, and pulse pressure, and with a lower HDL cholesterol. After mutual adjustment, in men and/or women, living in a low education neighborhood was related to a higher BMI, waist circumference, SBP, DBP, and resting heart rate, and to a lower HDL cholesterol. Additionally, men living alone had a lower BMI, waist circumference, total cholesterol, and glycaemia than men living in couple.

### Anthropometric risk factors

Men working in the Construction sector and in the Transport and communications sector had a higher BMI and tended to have a larger waist circumference (Table 1). On the opposite, men working in the Health and social work sector (the reference group) and in the Collective, social, and personal services sector had the lowest BMI and waist circumference.

**Table 1:** Associations with BMI and waist circumference estimated from multilevel regression models among men and women*

	BMI		Waist circumference	
	Men	Women	Men	Women
	β (95% CI)	β (95% CI)	β (95% CI)	β (95% CI)
**Work economic sector**				
Ref.: Health and social work				
Manufacturing industry	0.32 -0.65 – 1.30	-1.43 -2.82 – -0.03	-0.17 -3.01 – 2.66	-2.59 -5.87 – 0.69
Construction	1.50 0.43 – 2.57	-2.12 -5.49 – 1.24	3.62 0.52 – 6.73	-3.85 -11.76 – 4.05
Commercial, repair of motor vehicles and motorcycles	0.64 -0.34 – 1.62	-1.55 -2.85 – -0.26	1.39 -1.46 – 4.24	-3.47 -6.53 – -0.42
Hotels and restaurants	0.31 -0.73 – 1.34	-0.67 -2.26 – 0.92	0.23 -2.77 – 3.24	-0.02 -3.76 – 3.71
Transport and communications	1.15 0.13 – 2.18	-1.89 -3.46 – -0.33	2.44 -0.53 – 5.42	-4.45 -8.14 – -0.77
Financial activities	0.50 -0.49 – 1.50	-0.98 -2.37 – 0.41	0.20 -2.70 – 3.10	-3.08 -6.36 – 0.19
Real estate, renting and business services	0.58 -0.35 – 1.51	-1.17 -2.32 – -0.01	1.05 -1.65 – 3.75	-2.36 -5.10 – 0.36
Public administration	0.61 -0.58 – 1.81	-0.12 -1.55 – 1.31	1.45 -2.02 – 4.93	-0.96 -4.34 – 2.42
Education	0.59 -0.53 – 1.72	-0.95 -2.42 – 0.52	0.85 -2.40 – 4.11	-1.64 -5.13 – 1.83
Collective, social, and personal services	-0.13 -1.07 – 0.79	-1.99 -3.17 – -0.82	-1.04 -3.75 – 1.67	-4.35 -7.12 – -1.58
**Private (vs. public)**	0.09 -0.46 – 0.66	-0.68 -1.57 – 0.22	0.88 -0.74 – 2.52	-0.94 -3.04 – 1.16

*Models were adjusted for age, education, income, perceived financial strain, marital status, occupational status, and neighborhood level of education.

*Note.* BMI, body mass index; CI, confidence interval.

For women, the associations between work economic sectors and anthropometric variables were strikingly different. Women working in the Health and social work sector had the highest, not the lowest, BMI and waist circumference. Contrary to men, women working in the Transport and communications sector had among the lowest BMI and smallest waist circumference. Other work economic sectors associated with a lower BMI and waist circumference among women included the following sectors: Collective, social and personal services; Commercial and repair of vehicles; and Real estate, renting and business service.

### Blood pressure

Both men and women working in the Health and social work sector had the highest SBP (Table 2). On the opposite, both men and women working in the Collective, social and personal service sector had a relatively low SBP. Men working in the Hotels and restaurants sector also had a relatively low SBP.

**Table 2:** Associations with SBP, DBP, and pulse pressure estimated from multilevel regression models among men and women*

	SBP		DBP		Pulse pressure	
	Men	Women	Men	Women	Men	Women
	β (95% CI)	β (95% CI)	β (95% CI)	β (95% CI)	β (95% CI)	β (95% CI)
**Work economic sector**						
Ref.: Health and social work						
Manufacturing industry	-1.97 -6.02 – 2.08	-0.71 -5.89 – 4.46	1.37 -1.38 – 4.12	-3.09 -6.20 – 0.01	-3.33 -5.97 – -0.69	2.38 -1.11 – 5.88
Construction	-2.52 -6.96 – 1.91	-8.92 -21.33 – 3.48	1.39 -1.61 – 4.41	-5.20 -12.65 – 2.24	-3.91 -6.80 – -1.02	-3.71 -12.09 – 4.65
Commercial, repair of motor vehicles and motorcycles	-2.20 -6.27 – 1.87	-4.14 -8.96 – 0.67	0.87 -1.89 – 3.64	-5.16 -8.06 – -2.27	-2.98 -5.63 – -0.33	1.02 -2.23 – 4.27
Hotels and restaurants	-4.85 -9.15 – -0.56	-2.48 -8.36 – 3.40	0.29 -2.62 – 3.21	-2.08 -5.61 – 1.44	-5.15 -7.94 – -2.39	-0.39 -4.36 – 3.57
Transport and communications	-1.62 -5.87 – 2.61	-1.60 -7.42 – 4.21	1.43 -1.45 – 4.31	-4.74 -8.24 – -1.26	-3.05 -5.82 – -0.29	3.14 -0.78 – 7.07
Financial activities	-2.89 -7.03 – 1.25	-1.90 -7.05 – 3.24	0.96 -1.85 – 3.77	-2.02 -5.11 – 1.06	-3.86 -6.56 – -1.16	0.11 -3.35 – 3.58
Real estate, renting and business services	-3.24 -7.09 – 0.62	-2.01 -6.30 – 2.28	0.40 -2.21 – 3.02	-3.53 -6.10 – -0.95	-3.61 -6.13 – -1.10	1.52 -1.37 – 4.42
Public administration	-1.25 -6.22 – 3.72	-5.07 -10.37 – 0.23	0.31 -3.06 – 3.69	-2.79 -5.97 – 0.39	-1.62 -4.87 – 1.61	-2.27 -5.85 – 1.30
Education	-2.92 -7.57 – 1.72	-1.90 -7.39 – 3.59	0.34 -2.81 – 3.49	-1.87 -5.17 – 1.42	-3.29 -6.31 – -0.26	-0.02 -3.73 – 3.68
Collective, social and personal services	-3.88 -7.75 – -0.01	-6.17 -10.56 – -1.79	-0.12 -2.75 – 2.51	-4.90 -7.53 – -2.27	-3.77 -6.29 – -1.25	-1.27 -4.23 – 1.68
**Private (vs. public)**	0.60 -1.71 – 2.92	-0.92 -4.23 – 2.40	0.08 -1.49 – 1.65	1.32 -0.67 – 3.31	0.48 -1.02 – 1.99	-2.24 -4.48 – -0.01

*Models were adjusted for age, education, income, perceived financial strain, marital status, occupational status, antihypertensive medication use and neighborhood level of education.

*Note.* SBP, systolic blood pressure; DBP, diastolic blood pressure; CI, confidence interval.

No associations were documented between work economic sectors and DBP among men. Among women, a number of work economic sectors (especially Commercial and repair of vehicle; Collective, social, and personal services; Transport and communications; and Real estate, renting and business services) were associated with a lower DBP than in the Health and social work sector.

As a result of the different patterns of associations with SBP and DBP among men and women, no association was documented between work economic sectors and pulse pressure among women, while all economic sectors (especially Hotels and restaurants) were related to a lower pulse pressure than in the Health and social work sector among men. Women working in the private sector had a lower pulse pressure than those working in the public sector (private – public sector differences were documented for no other risk factor).

### Cholesterol

Work economic sectors were not associated with total cholesterol or LDL cholesterol among men and women (Table 3). Associations were documented between work economic sectors and HDL cholesterol, but only among men. Men working in the Health and social work sector had the highest HDL cholesterol, while the lowest HDL cholesterol levels were observed in the Hotels and restaurants sector and in the Education sector.

**Table 3:** Associations with total cholesterol, LDL cholesterol, and HDL cholesterol estimated from multilevel regression models among men and women*

	Total cholesterol		LDL cholesterol		HDL cholesterol	
	Men	Women	Men	Women	Men	Women
	β (95% CI)	β (95% CI)	β (95% CI)	β (95% CI)	β (95% CI)	β (95% CI)
**Work economic sector**						
Ref.: Health and social work						
Manufacturing industry	2.27 -9.03 – 13.58	-1.12 -12.28 – 10.03	3.71 -6.16 – 13.58	-3.08 -13.05 – 6.89	-1.74 -5.12 – 1.65	0.22 -3.82 – 4.26
Construction	8.05 -4.35 – 20.46	12.04 -14.48 – 38.55	9.00 -1.84 – 19.84	12.87 -10.75 – 36.50	-1.86 -5.58 – 1.85	0.89 -8.69 – 10.48
Commercial, repair of motor vehicles and motorcycles	1.26 -10.09 – 12.61	4.41 -5.95 – 14.79	3.03 -6.88 – 12.94	1.28 -8.00 – 10.57	-2.28 -5.68 – 1.12	2.53 -1.23 – 6.30
Hotels and restaurants	0.69 -11.33 – 12.72	12.84 -0.26 – 25.93	3.98 -6.52 – 14.49	8.83 -2.90 – 20.57	-4.98 -8.58 – -1.37	3.04 -1.72 – 7.80
Transport and communications	-4.53 -16.40 – 7.34	-0.45 -13.06 – 12.17	-1.64 -12.03 – 8.75	-3.25 -14.57 – 8.07	-3.52 -7.08 – 0.04	2.59 -1.99 – 7.18
Financial activities	4.41 -7.13 – 15.95	1.56 -9.67 – 12.81	6.87 -3.20 – 16.95	1.74 -8.31 – 11.80	-2.30 -5.76 – 1.15	-0.50 -4.58 – 3.58
Real estate, renting and business services	0.70 -10.06 – 11.46	4.84 -4.43 – 14.13	3.06 -6.34 – 12.45	2.45 -5.87 – 10.78	-2.61 -5.84 – 0.61	1.83 -1.54 – 5.21
Public administration	1.92 -12.09 – 15.92	-5.20 -16.69 – 6.29	1.38 -10.85 – 13.61	-2.01 -12.28 – 8.26	-2.55 -6.75 – 1.64	-2.46 -6.63 – 1.70
Education	-3.54 -16.43 – 9.33	4.04 -7.87 – 15.95	1.52 -9.72 – 12.77	3.70 -6.94 – 14.34	-3.87 -7.74 – -0.01	-0.77 -5.09 – 3.54
Collective, social and personal services	5.20 -5.57 – 15.97	-1.55 -11.01 – 7.89	7.30 -2.11 – 16.70	-2.51 -10.97 – 5.94	-2.89 -6.11 – 0.33	1.28 -2.14 – 4.72
**Private (vs. public)**	3.17 -3.29 – 9.64	-1.89 -9.11 – 5.32	2.16 -3.49 – 7.81	0.34 -6.10 – 6.77	-0.41 -2.35 – 1.52	-0.79 -3.40 – 1.82

*Models were adjusted for age, education, income, perceived financial strain, marital status, occupational status and neighborhood level of education.

*Note.* LDL, low-density lipoprotein; HDL, high-density lipoprotein; CI, confidence interval.

### Glycaemia

No association was documented with glycaemia among women (Table 4). Among men, those working in the Transportation and communications sector and in the Hotels and restaurants sector had the highest glycaemia levels while those working in the Health and social work sector had the lowest level.

**Table 4:** **Associations with glycaemia and resting heart rate estimated from multilevel regression models among men and women** ^*^

	Glycaemia		Resting heart rate	
	Men	Women	Men	Women
	β (95% CI)	β (95% CI)	β (95% CI)	β (95% CI)
**Work economic sector**				
Ref.: Health and social work				
Manufacturing industry	2.55 -1.32 – 6.43	0.65 -3.58 – 4.89	-1.36 -4.16 – 1.44	-2.22 -5.15 – 0.70
Construction	4.08 -0.17 – 8.33	1.28 -8.80 – 11.37	-1.84 -4.90 – 1.22	5.47 -1.59 – 12.53
Commercial, repair of motor vehicles and motorcycle	3.13 -0.75 – 7.02	1.56 -2.37 – 5.50	-0.83 -3.64 – 1.98	-2.14 -4.88 – 0.60
Hotels and restaurants	4.98 0.85 – 9.11	-0.66 -5.64 – 4.33	-1.39 -4.36 – 1.58	-3.68 -7.02 – -0.34
Transport and communications	6.30 2.23 – 10.37	-0.47 -5.24 – 4.28	-1.13 -4.07 – 1.80	-0.85 -4.14 – 2.43
Financial activities	3.34 -0.61 – 7.30	-0.25 -4.53 – 4.02	-1.08 -3.95 – 1.77	0.09 -2.83 – 3.02
Real estate, renting and business services	2.92 -0.76 – 6.61	0.26 -3.26 – 3.78	-0.95 -3.61 – 1.71	-0.22 -2.66 – 2.22
Public administration	0.87 -3.90 – 5.64	1.81 -2.55 – 6.18	0.32 -3.12 – 3.76	0.40 -2.62 – 3.42
Education	0.56 -3.88 – 4.99	-2.05 -6.58 – 2.47	-1.51 -4.73 – 1.70	0.72 -2.35 – 3.79
Collective, social and personal services	3.11 -0.57 – 6.80	-0.57 -4.16 – 3.01	-1.71 -4.38 – 0.96	-2.49 -4.96 – -0.02
**Private (vs. public)**	-0.39 -2.62 – 1.84	-1.31 -4.04 – 1.41	1.19 -0.42 – 2.80	0.36 -1.54 – 2.25

*Models were adjusted for age, education, income, perceived financial strain, marital status, occupational status, and neighborhood level of education.

*Note.* CI, confidence interval.

### Resting heart rate

No association was documented between work economic sectors and resting heart rate among men. As the only associations that were observed among women, those working in Hotels or restaurants and in the Collective, social, and personal services sector had the lowest resting heart rate (Table 4).

When work economic sectors were combined to distinguish between the secondary sector and the tertiary sector, no association was found with any of the cardiovascular risk factors among men and women (Additional file 6).

## Discussion

Because, as social class, work economic sectors may be seen as an upstream cause of diseases, and because knowledge on the relationships between work economic sectors and health may be useful to target Public health interventions, our aim was to investigate disparities between work economic sectors and most of the basic anthropometric and biological risk factors of cardiovascular diseases. In order to assess whether work economic sectors truly contributed to the variations in cardiovascular risk, our analyses were carefully adjusted for individual and neighborhood socioeconomic factors.

### Main findings

We found disparities in certain (but not all) cardiovascular risk factors between work economics sectors, and the patterns of such disparities were different in men and women and according to the risk factor.

Regarding similarities observed from one risk factor to the other, the Health and social work sector was found to be the most protective sector for BMI and waist circumference but also for glycaemia among men, while unfavorable profiles for these three risk factors among men were relatively consistently observed in the Construction sector and in the Transport and communications sector. Among men, the HDL cholesterol was also found to be the highest for those who were working in the Health and social work sector. However, this pattern did not apply to the other risk factors since no clear relationships were documented among men for total cholesterol, LDL cholesterol, and resting heart rate, and since, on the opposite, men working in the Health and social work sector showed the highest systolic BP and pulse pressure (rather than the lowest as for BMI, waist circumference, and glycaemia).

There were also discrepancies in the patterns of disparities between men and women. While the Health and social work sector was the most protective among men for different risk factors, women working in the Health and social work sector had the highest BMI, the largest waist circumference, and the most elevated systolic but also diastolic BP. On the opposite, the Commercial and repair of vehicles sector, the Transport and communication sector, and the Collective, social, and personal services sector were fairly consistently associated with a more favorable profile for these risk factors among women. Overall, it is not clear whether the higher SBP (and higher DBP among women) documented in the Health and social work sector may be due to the specific occupational risks of the sector, including strong constraints in the schedule and a high physical demand and job strain [22, 31].

Some of the disparities between work economic sectors may be due to differences in total energy intake or energy expenditure between work economic sectors. Regarding energy intake, a Japanese study [49] based on food records indicated that the daily average energy intake was higher in Craftsmen, in the Production process and construction personnel, and in Laborers (2432 kcal), in the service personnel (2444 kcal), in the professional and technical personnel (2174 kcal), and in the transport and communication personnel (2200 kcal). These data derived from selected individuals of a different country with markedly different dietary habits likely not represent the actual energy intake of the French subjects in the present study. If these findings on dietary habits are unable to explain, e.g., the higher BMI and larger waist circumference of men working in the Construction sector and Transportation and communications sector documented in our study, at least they show that energy intake can vary to a large extent between work economic sectors, as a potential explanation for the observed disparities. Similarly, our study does not empirically confirm that the trend of relationship between working in hotels and restaurants and higher total cholesterol and the relationship between working in this sector and lower HDL cholesterol documented among women is attributable to the specific dietary habits of these women.

Because work can be a major contributor to the total daily physical activity [50], an hypothesis may be that differences in physical activity levels between work economic sectors contribute to the reported economic disparities in cardiovascular risk factors. Based on this hypothesis, one would expect work economic sectors typically related with heavy manual work to be associated with a lower prevalence of cardiovascular risk factors, especially with a lower BMI and thinner waist circumference. However, we did not validate empirically this hypothesis, since for example men working in the Construction sector were found to have the highest BMI and largest waist circumference. This is in line with a previous study that indicated that, unlike recreational physical activity, occupational physical activity was not beneficial for cardiovascular risk factors, including those related to excess weight [51].

It should be noted that in addition to the social and physical characteristics of work conditions, the characteristics of the geographic environment around the typical locations of the worksites of each work economic sector may contribute to the reported associations.

Finally, also as an indication of the relevance of our examination of the different work economic sectors, we found that associations were lost when contrasting the secondary sector to the tertiary sector, which distinction was therefore not meaningful for cardiovascular risk factors.

### Strengths and limitations

Regarding study strengths, first, unlike previous studies on work economic sectors and cardiovascular risk factors, we examined in a comparative way a large panel of cardiovascular risk factors including BMI, waist circumference, SBP and DBP, pulse pressure, total cholesterol, HDL cholesterol, LDL cholesterol, glycaemia, and resting heart rate. While a large number of studies [5–9] have focused on differences in cardiovascular risk factors between occupational groups (as a *social* category), much less studies [7, 11, 28] have analyzed cardiovascular risk according to the work economic sector (as an *economic* variable). Our study is the very first to examine the relationships between work economic sectors and a large set of cardiovascular risk factors after controlling for individual and residential neighborhood socioeconomic variables. Another strength of our study is that information on the work economic sector was obtained through the linkage of administrative data to a population sample, leading to the presence of a very large number of workplaces (n = 3553 work establishments) and companies (n = 3005) in the sample, thus improving the generalizability of the findings.

Regarding study limitations, a first shortcoming of the work is related to the notion of work economic sector and operationalization of it that were used. It may be difficult to build a universal classification of work economic sectors because of the large difference in socioeconomic background between the countries [49]. In addition, it is sometimes problematic to classify a subject into only one work economic sector, since a number of companies and work establishments may be related to different economic sectors. Moreover, as the work life of workers is complex to assess and is the combination of a number of activities and circumstances, even within the same work economic sector, there may be notable variations in the lifestyles, in the magnitude of psychological stress, and in the intensity of physical activity (for a given occupation) between different territories, especially between different countries [11].

As a second limitation, the cross-sectional design of the study made it impossible to determine the direction of the causal effects involved in the associations reported (a poor cardiovascular health may differentially encourage to withdraw from professional activity according to the work economic sector, or may lead to seek for a job in specific sectors). Third, our sample recruited in preventive healthcare centers was not representative of the Paris Ile-de-France region [32]. However, a large panel of municipalities from the region was *a priori* selected to ensure the presence in the sample of people from all socioeconomic backgrounds. Moreover, the present analysis controlled for the individual and neighborhood factors that were found to influence participation in the study [52]. Fourth, the workplace was retrieved through the linkage of administrative information and we could not be formally sure that all the participants were still employed in the company and in the related work economic sector at the recruitment in the study. It may be expected, however, that participants changing from one company to another would tend to remain in the same work economic sector. Moreover, even if the participants were working in another work economic sector or were no longer working when they were enrolled in the study, they had been exposed to the work economic sector taken into account in the study the year prior to their recruitment (exposure to work economic sectors was measured homogeneously in the whole sample). Fifth and finally, the duration of employment in the work economic sector was not taken into account in the present study.

## Conclusion

In conclusion, the results of this study suggest that work economic sectors contribute to shape metabolic and cardiovascular parameters, even after adjustment for individual and neighborhood sociodemographic characteristics. The patterns of associations were found to vary, however, according to the risk factor examined and between men and women.

Overall, even if our study did not consider specific characteristics of work environments, the examination of the relationships between broad work economic sectors and a panel of cardiovascular risk factors is useful, in addition to the consideration of individual and residential characteristics, to determine profiles of populations to target Public health interventions and prevention efforts. As an example, while our previous work showed that having a low education, residing in neighborhoods with a low education and a low urbanicity degree, and shopping in hard discount supermarkets were all associated with a higher BMI and larger waist circumference [30, 36, 38], the present study further suggests that working in the Construction and in the Transport and communication sectors among males was also related to a larger body weight and fat, thus refining our knowledge on the profile of people at risk.

## Electronic supplementary material

Additional file 1: Table S1: **Tabulation of socioeconomic status by work economic sectors: % of participants in each category after excluding participants with missing information for the variable.** (DOCX 39 KB)

Additional file 2: **Associations between individual and neighborhood sociodemographic variables and BMI and waist circumference among men and women.** (DOCX 18 KB)

Additional file 3: **Associations between individual and neighborhood sociodemographic variables and SBP, DBP, and pulse pressure among men and women.** (DOCX 21 KB)

Additional file 4: **Associations between individual and neighborhood sociodemographic variables and total cholesterol, LDL cholesterol, and HDL cholesterol among men and women.** (DOCX 19 KB)

Additional file 5: **Associations between individual and neighborhood sociodemographic variables and glycaemia and resting heart rate among men and women.** (DOCX 18 KB)

Additional file 6: **Associations between working in the secondary rather than in the tertiary sector and cardiovascular risk factors estimated from multilevel regression models among men and women.** (DOCX 16 KB)

## Abbreviations

BMI: Body mass index
SBP: Systolic blood pressure
DBP: Diastolic blood pressure
HDL: High-density lipoprotein
LDL: Low-density lipoprotein
CI: Confidence interval
CNAV: National Old Age Insurance System
Insee: French National Institute of Statistics and Economic Studies.

## References

[1] Yusuf S, Islam S, Chow CK, Rangarajan S, Dagenais G, Diaz R, Gupta R, Kelishadi R, Iqbal R, Avezum A, Kruger A, Kutty R, Lanas F, Lisheng L, Wei L, Lopez-Jaramillo P, Oguz A, Rahman O, Swidan H, Yusoff K, Zatonski W, Rosengren A, Teo KK, Prospective Urban Rural Epidemiology (PURE) Study Investigators (2011). Use of secondary prevention drugs for cardiovascular disease in the community in high-income, middle-income, and low-income countries (the PURE Study): a prospective epidemiological survey. Lancet.

[2] Gupta R, Kaul V, Agrawal A, Guptha S, Gupta VP (2010). Cardiovascular risk according to educational status in India. Prev Med.

[3] Reddy KS, Prabhakaran D, Jeemon P, Thankappan KR, Joshi P, Chaturvedi V, Ramakrishnan L, Ahmed F (2007). Educational status and cardiovascular risk profile in Indians. Proc Natl Acad Sci U S A.

[4] Jeemon P, Prabhakaran D, Huffman MD, Ramakrishnan L, Goenka S, Thankappan KR, Mohan V, Joshi PP, Mohan BV, Ahmed F, Ramanathan M, Ahuja R, Chaturvedi V, Lloyd-Jones DM, Reddy KS, Sentinel Surveillance in Industrial Populations Study Group (2011). Distribution of 10-year and lifetime predicted risk for cardiovascular disease in the Indian Sentinel Surveillance Study population (cross-sectional survey results). BMJ Open.

[5] Frommer MS, Edye BV, Mandryk JA, Grammeno GL, Berry G, Ferguson DA (1986). Systolic blood pressure in relation to occupation and perceived work stress. Scand J Work Environ Health.

[6] Hu G, Jousilahti P, Antikainen R, Tuomilehto J (2007). Occupational, commuting, and leisure-time physical activity in relation to cardiovascular mortality among finnish subjects with hypertension. Am J Hypertens.

[7] Leigh JP, Du J (2009). Hypertension and occupation among seniors. J Occup Environ Med.

[8] Schnall PL, Landsbergis PA, Baker D (1994). Job strain and cardiovascular disease. Annu Rev Public Health.

[9] Tsutsumi A, Kayaba K, Tsutsumi K, Igarashi M (2001). Association between job strain and prevalence of hypertension: a cross sectional analysis in a Japanese working population with a wide range of occupations: the Jichi Medical School cohort study. Occup Environ Med.

[10] Kaplan GA, Keil JE (1993). Socioeconomic factors and cardiovascular disease: a review of the literature. Circulation.

[11] Takashima Y, Yoshida M, Kokaze A, Orido Y, Tsugane S, Ishikawa M, Takeuchi Y, Takagi Y, Tanaka N, Watanabe S, Akamatsu T (1998). Relationship of occupation to blood pressure among middle-aged Japanese men–the significance of the differences in body mass index and alcohol consumption. J Epidemiol.

[12] Haglund BJ (1985). Geographical and socioeconomic distribution of high blood pressure and borderline high blood pressure in a Swedish rural county. Scand J Soc Med.

[13] Covey LS, Wynder EL (1981). Smoking habits and occupational status. J Occup Med.

[14] Dobson AJ, Gibberd RW, Leeder SR, O'Connell DL (1985). Occupational differences in ischemic heart disease mortality and risk factors in Australia. Am J Epidemiol.

[15] Simons LA, Simons J, Magnus P, Bennett SA (1986). Education level and coronary risk factors in Australians. Med J Aust.

[16] Helmert U, Shea S, Herman B, Greiser E (1990). Relationship of social class characteristics and risk factors for coronary heart disease in West Germany. Public Health.

[17] Kritz-Silverstein D, Wingard DL, Barrett-Connor E (1992). Employment status and heart disease risk factors in middle-aged women: the Rancho Bernardo Study. Am J Public Health.

[18] Wilson TW, Kaplan GA, Kauhanen J, Cohen RD, Wu M, Salonen R, Salonen JT (1993). Association between plasma fibrinogen concentration and five socioeconomic indices in the Kuopio Ischemic Heart Disease Risk Factor Study. Am J Epidemiol.

[19] Medalie JH, Papier C, Herman JB, Goldbourt U, Tamir S, Neufeld HN, Riss E (1974). Diabetes mellitus among 10,000 adult men. I Five-year incidence and associated variables Isr J Med Sci.

[20] Milczarek M, Schneider E, González ER (2009). Stress at Work. Volume 9. Edited by Report ERO.

[21] Holman D, McClelland C (2011). Job Quality in Growing and Declining Economic Sectors of the EU. Work and Life Quality in new & Growing Jobs.

[22] DARES, Direction de l’animation de la recherche des études et des statistiques (2009). Conditions de travail et santé.

[23] Klein T, Long K (2013). Working conditions, work organization and use of ICT as business. Center for Strategic Analysis, Department Labour Employment.

[24] Sandor E (2009). European Company Survey 2009. Part-time work in Europe. European Fundation for the Improvement of Living and Working Conditions.

[25] Lauzeville D, Marchand JL, Ferrand M (2009). Consommation de tabac par catégorie socioprofessionnelle et secteur d'activité: outil méthodologique pour l'épidémiologie.

[26] Beck F (2012). Résultats du Baromètre Santé 2010 - Liens entre usages de substances psychoactives (SPA) et milieu professionnel. Enquêtes et analyses statistiques.

[27] Goffette C (2012). Le contexte social du tabagisme: Le rôle de l'environnement familial et professionnel.

[28] Proper KI, Hildebrandt VH (2010). Overweight and obesity among Dutch workers: differences between occupational groups and sectors. Int Arch Occup Environ Health.

[29] Hasson D, Von Thiele SU, Lindfors P (2009). Self-rated health and allostatic load in women working in two occupational sectors. J Health Psychol.

[30] Leal C, Bean K, Thomas F, Chaix B (2011). Are associations between neighborhood socioeconomic characteristics and body mass index or waist circumference based on model extrapolations?. Epidemiology.

[31] Chaix B, Bean K, Leal C, Thomas F, Havard S, Evans D, Jego B, Pannier B (2010). Individual/neighborhood social factors and blood pressure in the RECORD Cohort Study: which risk factors explain the associations?. Hypertension.

[32] Chaix B, Billaudeau N, Thomas F, Havard S, Evans D, Kestens Y, Bean K (2011). Neighborhood effects on health: correcting bias from neighborhood effects on participation. Epidemiology.

[33] Chaix B, Jouven X, Thomas F, Leal C, Billaudeau N, Bean K, Kestens Y, Jego B, Pannier B, Danchin N (2011). Why socially deprived populations have a faster resting heart rate: impact of behaviour, life course anthropometry, and biology–the RECORD Cohort Study. Soc Sci Med.

[34] Chaix B, Kestens Y, Bean K, Leal C, Karusisi N, Meghiref K, Burban J, Fon Sing M, Perchoux C, Thomas F, Merlo J, Pannier B (2012). Cohort profile: residential and non-residential environments, individual activity spaces and cardiovascular risk factors and diseases–the RECORD Cohort Study. Int J Epidemiol.

[35] Havard S, Reich BJ, Bean K, Chaix B (2011). Social inequalities in residential exposure to road traffic noise: an environmental justice analysis based on the RECORD Cohort Study. Occup Environ Med.

[36] Chaix B, Bean K, Daniel M, Zenk SN, Kestens Y, Charreire H, Leal C, Thomas F, Karusisi N, Weber C, Oppert JM, Simon C, Merlo J, Pannier B (2012). Associations of supermarket characteristics with weight status and body fat: a multilevel analysis of individuals within supermarkets (RECORD study). PLoS One.

[37] Karusisi N, Bean K, Oppert JM, Pannier B, Chaix B (2012). Multiple dimensions of residential environments, neighborhood experiences, and jogging behavior in the RECORD Study. Prev Med.

[38] Leal C, Bean K, Thomas F, Chaix B (2012). Multicollinearity in associations between multiple environmental features and body weight and abdominal fat: using matching techniques to assess whether the associations are separable. Am J Epidemiol.

[39] Karusisi N, Thomas F, Meline J, Chaix B (2013). Spatial accessibility to specific sport facilities and corresponding sport practice: the RECORD Study. Int J Behav Nutr Phys Act.

[40] INSEE (2011). IRIS—Definition. Paris, France: National Institute of Statistics and Economic Studies.

[41] *Cardiovascular disease*. [http://www.who.int/mediacentre/factsheets/fs317/en/]

[42] Thomas F, Bean K, Pannier B, Oppert JM, Guize L, Benetos A (2005). Cardiovascular mortality in overweight subjects: the key role of associated risk factors. Hypertension.

[43] WHO (2000). Obesity: preventing and managing the global epidemic. Report of a WHO consultation. World Health Organ Tech Rep Ser.

[44] Benetos A, Thomas F, Pannier B, Bean K, Jego B, Guize L (2008). All-cause and cardiovascular mortality using the different definitions of metabolic syndrome. Am J Cardiol.

[45] Pannier B, Thomas F, Bean K, Jego B, Benetos A, Guize L (2008). The metabolic syndrome: similar deleterious impact on all-cause mortality in hypertensive and normotensive subjects. J Hypertens.

[46] Benetos A, Rudnichi A, Thomas F, Safar M, Guize L (1999). Influence of heart rate on mortality in a French population: role of age, gender, and blood pressure. Hypertension.

[47] Chaix B, Simon C, Charreire H, Thomas F, Kestens Y, Karusisi N, Vallee J, Oppert JM, Weber C, Pannier B (2014). The environmental correlates of overall and neighborhood based recreational walking (a cross-sectional analysis of the RECORD Study). Int J Behav Nutr Phys Act.

[48] Chaix B, Leal C, Evans D (2010). Neighborhood-level confounding in epidemiologic studies: unavoidable challenges, uncertain solutions. Epidemiology.

[49] Takashima Y, Iwase Y, Yoshida M, Kokaze A, Takagi Y, Taubono Y, Tsugane S, Takahashi T, Iitoi Y, Akabane M, Watanabe S, Akamatsu T (1998). Relationship of food intake and dietary patterns with blood pressure levels among middle-aged Japanese men. J Epidemiol.

[50] Proper KI, Hildebrandt VH (2006). Physical activity among Dutch workers–differences between occupations. Prev Med.

[51] Oppert JM, Thomas F, Charles MA, Benetos A, Basdevant A, Simon C (2006). Leisure-time and occupational physical activity in relation to cardiovascular risk factors and eating habits in French adults. Public Health Nutr.

[52] Chaix B, Evans D, Merlo J, Suzuki E (2012). Commentary: Weighing up the dead and missing: reflections on inverse-probability weighting and principal stratification to address truncation by death. Epidemiology.

[CR53] The pre-publication history for this paper can be accessed here:http://www.biomedcentral.com/1471-2458/14/750/prepub

