# Supplementary material for: Work economic sectors and cardiovascular risk factors: cross-sectional analysis based on the RECORD Study
Source: BMC Public Health. 2014 Jul 24;14:750. doi: 10.1186/1471-2458-14-750 (PMC4137071; doi:10.1186/1471-2458-14-750)
Supplement: Supplementary file 1 — Additional file 1: Table S1: Tabulation of socioeconomic status by work economic sectors: % of participants in each category after excluding participants with missing information for the variable. (DOCX 39 KB) [file 12889_2014_6938_MOESM1_ESM.docx]

| **Additional Table 1.** Tabulation of socioeconomic status by work economic sectors: % of participants in each category after excluding participants with missing information for the variable | | | | | | | | | | | |
| --- | --- | --- | --- | --- | --- | --- | --- | --- | --- | --- | --- |
|  | **Health and social work** | **Manufacturing industry** | **Construction** | **Commercial, repair of motor vehicles and motorcycles** | **Hotels and restaurants** | **Transport and communications** | **Financial activities** | **Real estate, renting and business services** | **Public**  **administration** | **Education** | **Collective, social, and personal services** |
| **Variables** | **(n =180)** | **(n =496)** | **(n =173)** | **(n =508)** | **(n =239)** | **(n =256)** | **(n =408)** | **(n =1332)** | **(n =128)** | **(n =142)** | **(n =498)** |
| **Age**  30-44  45-59  60-79 | 39  51  10 | 37  52  11 | 47  47  6 | 46  45  9 | 55  37  8 | 49  44  7 | 34  58  8 | 49  43  8 | 39  48  13 | 42  37  21 | 46  46  8 |
| **Individual education**  Low  Medium-Low  Medium-High  High | 12  33  28  27 | 4  24  27  45 | 18  43  25  14 | 8  25  34  33 | 19  37  31  13 | 6  18  31  45 | 2  17  27  54 | 8  15  28  49 | 8  32  33  27 | 4  14  28  54 | 7  20  33  40 |
| **Occupation**  Blue-collar  Low white-collar  Intermediate  High white-collar | 16  56  13  15 | 13  19  10  58 | 54  13  11  22 | 16  32  9  43 | 38  47  4  11 | 15  22  9  54 | 2  26  4  68 | 13  30  6  51 | 15  55  6  24 | 6  51  6  37 | 11  47  9  33 |
| **Household income**  Low  Medium-low  Medium-high  High | 51  23  17  9 | 17  24  25  34 | 39  29  19  13 | 26  29  21  24 | 52  25  13  10 | 23  20  25  32 | 9  19  32  40 | 28  21  22  29 | 47  27  16  10 | 39  25  15  21 | 21  28  27  24 |
| **Living alone** | 41 | 11 | 21 | 26 | 33 | 26 | 7 | 30 | 49 | 37 | 32 |
| **Perceived financial strain** | 32 | 10 | 16 | 18 | 31 | 13 | 28 | 18 | 36 | 26 | 16 |
| **Antihypertensive medication use** | 10 | 6 | 8 | 5 | 5 | 6 | 7 | 5 | 14 | 6 | 4 |
| **Educational level of local residents**  Low  Medium-low  Medium-high  High | 41  27  17  15 | 23  23  26  28 | 53  21  16  10 | 29  20  27  24 | 33  26  21  20 | 23  23  25  29 | 12  21  28  39 | 25  21  26  28 | 35  26  24  15 | 18  22  24  36 | 18  22  31  29 |
|  | | | | | | | | | | | |
