# Supplementary material for: Work economic sectors and cardiovascular risk factors: cross-sectional analysis based on the RECORD Study
Source: BMC Public Health. 2014 Jul 24;14:750. doi: 10.1186/1471-2458-14-750 (PMC4137071; doi:10.1186/1471-2458-14-750)
Supplement: Supplementary file 2 — Additional file 2: Associations between individual and neighborhood sociodemographic variables and BMI and waist circumference among men and women. (DOCX 18 KB) [file 12889_2014_6938_MOESM2_ESM.docx]

| **Additional file 2** Associations between individual and neighborhood sociodemographic variables and BMI and waist circumference among men and women. | | | | | |
| --- | --- | --- | --- | --- | --- |
|  | **BMI** | | | **Waist circumference** | |
|  | **Men** | **Women** | | **Men** | **Women** |
|  | **β (95% CI)** | | **β (95% CI)** | **β (95% CI)** | **β (95% CI)** |
| **Age (vs. 30 to 44)** |  |  | |  |  |
| 45 to 59 | 0.77 0.51 – 1.03 | 0.91 0.36 – 1.46 | | 3.77 3.01 – 4.53 | 2.62 1.32 – 3.92 |
| 60 to 79 | 1.21 0.74 – 1.68 | 1.97 1.04 – 2.90 | | 5.87 4.51 – 7.24 | 5.66 3.47 – 7.85 |
| **Individual education (vs. high education)** |  |  | |  |  |
| Medium-high education | 0.50 0.17 – 0.83 | 0.18 -0.51 – 0.87 | | 1.22 0.26 – 2.18 | 0.36 -1.26 – 1.99 |
| Medium-low education | 0.61 0.20 – 1.02 | 1.36 0.53 – 2.20 | | 1.98 0.79 – 3.17 | 3.09 1.12 – 5.06 |
| Low education | 0.56 -0.02 – 1.15 | 2.68 1.53 – 3.83 | | 0.92 -0.79 – 2.64 | 5.05 2.35 – 7.75 |
| **Household income (vs. high income)** |  |  | |  |  |
| Medium-high income | -0.13 -0.48 – 0.22 | 0.17 -0.63 – 0.97 | | -0.18 -1.21 – 0.84 | 0.17 -1.72 – 2.05 |
| Medium-low income | -0.32 -0.69 – 0.05 | 0.96 0.15 – 1.78 | | -0.58 -1.67 – 0.51 | 1.49 -0.42 – 3.40 |
| Low income | -0.66 -1.11 – -0.22 | 0.62 -0.26 – 1.50 | | -0.98 -2.27 – 0.31 | 1.31 -0.76 – 3.38 |
| **Perceived financial strain** | 0.51 0.13 – 0.90 | 1.11 0.43 – 1.78 | | 1.03 -0.09 – 2.15 | 2.59 0.99 – 4.18 |
| **Living alone (vs. as a couple)** | -0.92 -1.22 – -0.62 | -0.20 -0.73 – 0.33 | | -2.13 -3.01 – -1.25 | -0.92 -2.17 – 0.32 |
| **Occupational status (vs. high white-collar)** |  |  | |  |  |
| Intermediate | -0.20 -0.74 – 0.33 | -0.05 -1.16 – 1.06 | | -0.98 -2.53 – 0.58 | -0.77 -3.39 – 1.85 |
| Low-white collar | 0.09 -0.28 – 0.46 | -0.32 -1.03 – 0.38 | | -0.08 -1.16 – 1.01 | -0.22 -1.87 – 1.43 |
| Blue-collar | -0.45 -0.93 – 0.02 | -0.14 -1.41 – 1.13 | | -2.08 -3.46 – -0.71 | -0.53 -3.52 – 2.46 |
| **Residential education level (vs. high)** |  |  | |  |  |
| Medium-high | 0.13 -0.20 – 0.47 | -0.15 -0.87 – 0.57 | | -0.31 -1.29 – 0.68 | -0.54 -2.24 – 1.16 |
| Medium-low | 0.26 -0.10 – 0.63 | 0.34 -0.41 – 1.09 | | 0.28 -0.78 – 1.35 | 0.14 -1.64 – 1.91 |
| Low | 1.01 0.62 – 1.39 | 1.78 0.98 – 2.58 | | 1.55 0.43 – 2.67 | 3.59 1.70 – 5.48 |
| *Note.* CI, confidence interval; BMI, Body Mass Index. | | | | | |
