# Supplementary material for: Work economic sectors and cardiovascular risk factors: cross-sectional analysis based on the RECORD Study
Source: BMC Public Health. 2014 Jul 24;14:750. doi: 10.1186/1471-2458-14-750 (PMC4137071; doi:10.1186/1471-2458-14-750)
Supplement: Supplementary file 3 — Additional file 3: Associations between individual and neighborhood sociodemographic variables and SBP, DBP, and pulse pressure among men and women. (DOCX 21 KB) [file 12889_2014_6938_MOESM3_ESM.docx]

| **Additional file 3** Associations between individual and neighborhood sociodemographic variables and SBP, DBP, and pulse pressure among men and women. | | | | | | |
| --- | --- | --- | --- | --- | --- | --- |
|  | **SBP** | | **DBP** | | **Pulse pressure** | |
|  | **Men** | **Women** | **Men** | **Women** | **Men** | **Women** |
|  | **β (95% IC)** | **β (95% IC)** | **β (95% IC)** | **β (95% IC)** | **β (95% IC)** | **β (95% IC)** |
| **Age (vs. 30 to 44)** |  |  |  |  |  |  |
| 45 to 59 | 5.26 4.16 – 6.35 | 7.86 5.80 – 9.93 | 5.26 4.51 – 6.00 | 2.89 1.66 – 4.14 | 0.03 -0.68 – 0.74 | 4.96 3.57 – 6.36 |
| 60 to 79 | 10.02 8.04 – 11.99 | 14.06 10.56 – 17.57 | 5.67 4.32 – 7.01 | 4.86 2.76 – 6.97 | 4.34 3.05 – 5.63 | 9.20 6.83 – 11.56 |
| **Individual education (vs. high education)** |  |  |  |  |  |  |
| Medium-high education | 1.34 -0.03 – 2.71 | -0.20 -2.78 – 2.37 | 0.87 -0.05 – 1.81 | -0.40 -1.95 – 1.14 | 0.48 -0.41 – 1.37 | 0.19 -1.54 – 1.93 |
| Medium-low education | 3.02 1.32 – 4.73 | 2.26 -0.86 – 5.39 | 1.33 0.18 – 2.49 | 0.86 -1.01 – 2.74 | 1.73 0.62 – 2.84 | 1.40 -0.70 – 3.51 |
| Low education | 2.83 0.364 – 5.29 | 3.76 -0.50 – 8.01 | 0.60 -1.07 – 2.27 | 2.64 0.09 – 5.20 | 2.21 0.60 – 3.82 | 1.11 -1.76 – 3.98 |
| **Household income (vs. high income)** |  |  |  |  |  |  |
| Medium-high income | -1.74 -3.20 – -0.27 | -1.41 -4.40 – 1.58 | -0.41 -1.41 – 0.57 | -0.22 -2.02 – 1.57 | -1.37 -2.32 – -0.41 | -1.18 -3.20 – 0.83 |
| Medium-low income | 0.08 -1.48 – 1.64 | 0.06 -2.96 – 3.07 | 0.16 -0.90 – 1.22 | 1.06 -0.74 – 2.87 | -0.15 -1.17 – 0.87 | -1.00 -3.04 – 1.02 |
| Low income | 0.38 -1.47 – 2.23 | -0.41 -3.69 – 2.88 | 0.69 -0.57 – 1.94 | 0.24 -1.72 – 2.21 | -0.39 -1.60 – 0.81 | -0.65 -2.87 – 1.56 |
| **Perceived financial strain** | -0.84 -2.45 – 0.76 | 0.77 -1.75 – 3.29 | -0.02 -1.11 – 1.07 | -0.50 -2.01 – 1.01 | -0.82 -1.86 – 0.23 | 1.27 -0.43 – 2.97 |
| **Living alone (vs. as a couple)** | -0.26 -1.51 – 0.99 | 1.12 -0.85 – 3.08 | -0.11 -0.97 – 0.74 | 0.97 -0.21 – 2.14 | -0.17 -0.99 – 0.64 | 0.15 -1.17 – 1.47 |
| **Occupational status (vs. white-collar)** |  |  |  |  |  |  |
| Intermediate | 0.21 -2.02 – 2.43 | 1.71 -2.41 – 5.83 | 0.02 -1.48 – 1.53 | 1.33 -1.13 – 3.81 | 0.19 -1.25 – 1.64 | 0.37 -2.40 – 3.15 |
| Low-white collar | -0.03 -1.58 – 1.52 | 1.62 -1.00 – 4.24 | -0.94 -1.99 – 0.11 | 1.02 -0.55 – 2.59 | 0.94 -0.07 – 1.96 | 0.60 -1.16 – 2.37 |
| Blue-collar | -0.00 -1.97 – 1.96 | 6.54 1.82 – 11.27 | -0.45 -1.79 – 0.88 | 3.67 0.83 – 6.51 | 0.56 -0.72 – 1.85 | 2.87 -0.31 – 6.06 |
| **Antihypertensive medication** | 10.47 8.04 – 12.89 | 12.86 9.12 – 16.60 | 5.22 3.58 – 6.87 | 7.25 5.01 – 9.49 | 5.22 3.64 – 6.79 | 5.61 3.09 – 8.13 |
| **Residential education level (vs. high)** |  |  |  |  |  |  |
| Medium-high | 1.73 0.31 – 3.15 | -1.84 -4.52 – 0.83 | 1.07 0.12 – 2.02 | -1.21 -2.82 – 0.39 | 0.69 -0.21 – 1.61 | -0.63 -2.43 – 1.17 |
| Medium -low | 1.83 0.30 – 3.35 | -0.93 -3.73 – 1.86 | 0.60 -0.42 – 1.63 | -0.68 -2.36 – 0.99 | 1.28 0.30 – 2.27 | -0.25 -2.13 – 1.63 |
| Low | 2.41 0.80 – 4.02 | 0.64 -2.34 – 3.61 | 1.46 0.37 – 2.54 | 1.49 -0.29 – 3.27 | 0.93 -0.10 – 1.97 | -0.85 -2.86 – 1.15 |
| *Note.* SBP, diastolic blood pressure; DBP, systolic blood pressure; CI, confidence interval. | | | | | | |
