# Supplementary material for: Work economic sectors and cardiovascular risk factors: cross-sectional analysis based on the RECORD Study
Source: BMC Public Health. 2014 Jul 24;14:750. doi: 10.1186/1471-2458-14-750 (PMC4137071; doi:10.1186/1471-2458-14-750)
Supplement: Supplementary file 4 — Additional file 4: Associations between individual and neighborhood sociodemographic variables and total cholesterol, LDL cholesterol, and HDL cholesterol among men and women. (DOCX 19 KB) [file 12889_2014_6938_MOESM4_ESM.docx]

| **Additional file 4** Associations between individual and neighborhood sociodemographic variables and total cholesterol, LDL cholesterol, and HDL cholesterol among men and women. | | | | | | |
| --- | --- | --- | --- | --- | --- | --- |
|  | **Total cholesterol** | | **LDL cholesterol** | | **HDL cholesterol** | |
|  | **Men** | **Women** | **Men** | **Women** | **Men** | **Women** |
|  | **β (95% IC)** | **β (95% IC)** | **β (95% IC)** | **β (95% IC)** | **β (95% IC)** | **β (95% IC)** |
| **Age (vs. 30 to 44)** |  |  |  |  |  |  |
| 45 to 59 | 11.37 8.37 – 14.37 | 25.15 20.72 – 29.59 | 8.90 6.27 – 11.52 | 19.63 15.67 – 23.58 | 0.94 0.04 – 1.84 | 3.88 2.28 – 5.49 |
| 60 to 79 | 9.78 4.26 – 15.30 | 37.44 29.98 – 44.91 | 5.94 1.11 – 10.77 | 29.57 22.90 – 36.24 | 3.02 1.36 – 4.68 | 5.10 2.39 – 7.80 |
| **Individual education (vs. high education)** |  |  |  |  |  |  |
| Medium-high education | 5.17 1.38 – 8.97 | 5.83 0.30 – 11.37 | 4.15 0.83 – 7.47 | 5.66 0.72 – 10.61 | -1.00 -2.14 – 0.13 | -1.48 -3.48 – 0.52 |
| Medium-low education | 6.97 2.28 – 11.66 | 5.63 -1.08 – 12.34 | 3.97 -0.13 – 8.07 | 7.05 1.06 – 13.05 | 0.27 -1.13 – 1.68 | -3.50 -5.93 – -1.07 |
| Low education | 2.13 -4.59 – 8.87 | 1.74 -7.47 – 10.96 | 1.26 -4.67 – 7.19 | 4.98 -3.24 – 13.21 | -0.80 -2.83 – 1.23 | -5.60 -8.94 – -2.26 |
| **Household income (vs. high income)** |  |  |  |  |  |  |
| Medium-high income | -0.10 -4.15 – 3.95 | -2.62 -9.05 – 3.80 | 0.17 -3.37 – 3.71 | -0.40 -6.12 – 5.32 | 0.61 -0.60 – 1.83 | -1.69 -4.01 – 0.63 |
| Medium-low income | -0.47 -4.81 – 3.86 | -2.91 -9.43 – 3.59 | -0.57 -4.36 – 3.22 | -0.40 -6.21 – 5.40 | -0.03 -1.34 – 1.26 | -1.73 -4.09 – 0.62 |
| Low income | -2.62 -7.74 – 2.49 | -4.38 -11.47 – 2.71 | 2.97 -7.45 – 1.50 | -0.84 -7.16 – 5.49 | -0.39 -1.92 – 1.14 | -2.86 -5.42 – -0.29 |
| **Perceived financial strain** | -2.90 -7.34 – 1.52 | 1.87 -3.64 – 7.38 | -2.05 -5.94 – 1.83 | 2.87 -2.04 – 7.79 | -2.29 -3.62 – -0.96 | -2.10 -4.09 – -0.10 |
| **Living alone (vs. as a couple)** | -4.10 -7.56 – -0.63 | -1.44 -5.69 – 2.81 | -3.99 -7.02 – -0.96 | -2.48 -6.28 – 1.31 | 0.60 -0.44 – 1.64 | 0.71 -0.83 – 2.25 |
| **Occupational status (vs. white-collar)** |  |  |  |  |  |  |
| Intermediate | -2.29 -8.42 – 3.83 | -1.49 -10.34 – 7.36 | -0.31 -5.67 – 5.03 | -3.61 -11.49 – 4.26 | 0.32 -1.51 – 2.16 | 1.29 -1.89 – 4.49 |
| Low-white collar | -1.26 -5.56 – 3.03 | -3.66 -9.32 – 1.99 | -1.33 -5.09 – 2.43 | -4.10 -9.14 – 0.94 | 0.23 -1.06 – 1.52 | 0.34 -1.70 – 2.39 |
| Blue-collar | -2.71 -8.13 – 2.71 | 2.18 -8.03 – 12.40 | -2.69 -7.44 – 2.04 | -0.13 -9.26 – 9.00 | 1.80 0.17 – 3.43 | 2.63 -1.07 – 6.34 |
| **Residential education level (vs. high)** |  |  |  |  |  |  |
| Medium-high | 1.95 -1.93 – 5.85 | -1.08 -6.92 – 4.75 | 1.94 -1.46 – 5.35 | -0.12 -5.25 – 5.01 | -0.24 -1.43 – 0.94 | -0.69 -2.77 – 1.39 |
| Medium -low | -0.83 -5.04 – 3.37 | -2.81 -8.94 – 3.31 | 0.22 -3.47 – 3.91 | -0.62 -6.03 – 4.77 | -0.73 -2.01 – 0.55 | -1.37 -3.57 – 0.81 |
| Low | -3.72 -8.17 – 0.71 | -2.86 -9.33 – 3.60 | -1.18 -5.07 – 2.70 | -0.13 -5.83 – 5.58 | -1.63 -2.98 – -0.28 | -2.54 -4.86 – -0.23 |
| *Note.* LDL, low-density lipoprotein; HDL, high-density lipoprotein; CI, confidence interval. | | | | | | |
