# Supplementary material for: Work economic sectors and cardiovascular risk factors: cross-sectional analysis based on the RECORD Study
Source: BMC Public Health. 2014 Jul 24;14:750. doi: 10.1186/1471-2458-14-750 (PMC4137071; doi:10.1186/1471-2458-14-750)
Supplement: Supplementary file 5 — Additional file 5: Associations between individual and neighborhood sociodemographic variables and glycaemia and resting heart rate among men and women. (DOCX 18 KB) [file 12889_2014_6938_MOESM5_ESM.docx]

| **Additional file 5** Associations between individual and neighborhood sociodemographic variables and glycaemia and resting heart rate among men and women. | | | | | |
| --- | --- | --- | --- | --- | --- |
|  | **Glycaemia** | | | **Resting heart rate** | |
|  | **Men** | **Women** | | **Men** | **Women** |
|  | **β (95% CI)** | | **β (95% CI)** | **β (95% CI)** | **β (95% CI)** |
| **Age (vs. 30 to 44)** |  |  | |  |  |
| 45 to 59 | 5.00 3.97 – 6.04 | 4.75 3.07 – 6.44 | | 1.15 0.40 – 1.91 | -0.37 -1.54 – 0.78 |
| 60 to 79 | 7.24 5.33 – 9.14 | 7.83 5.00 – 10.66 | | 1.73 0.38 – 3.09 | -0.05 -2.01 – 1.89 |
| **Individual education (vs. high education)** |  |  | |  |  |
| Medium-high education | 0.31 -0.99 – 1.62 | -0.44 -2.55 – 1.65 | | 1.14 0.19 – 2.09 | -0.27 -1.73 – 1.19 |
| Medium-low education | 1.29 -0.32 – 2.91 | -0.23 -2.78 – 2.31 | | 1.64 0.46 – 2.81 | -0.97 -2.74 – 0.79 |
| Low education | 0.75 -1.57 – 3.08 | -0.63 -4.13 – 2.86 | | 0.19 -1.50 – 1.90 | 1.54 -0.89 – 3.98 |
| **Household income (vs. high income)** |  |  | |  |  |
| Medium-high income | -1.27 -2.67 – 0.12 | -2.62 -5.05 – -0.19 | | -0.12 -1.13 – 0.89 | -1.36 -3.04 – 0.33 |
| Medium-low income | -1.07 -2.57 – 0.42 | -1.95 -4.43 – 0.53 | | 0.80 -0.27 – 1.89 | -0.80 -2.51 – 0.90 |
| Low income | -1.68 -3.45 – 0.08 | -2.26 -4.96 – 0.43 | | 2.59 1.31 – 3.87 | 0.68 -1.17 – 2.53 |
| **Perceived financial strain** | 0.21 -1.31 – 1.74 | -0.63 -4.13 – 2.86 | | 0.04 -1.07 – 1.15 | -0.86 -2.29 – 0.57 |
| **Living alone (vs. as a couple)** | -1.36 -2.55 – -0.16 | 0.24 -1.37 – 1.86 | | 0.57 -0.29 – 1.44 | -0.27 -1.39 – 0.84 |
| **Occupational status (vs. high white-collar)** |  |  | |  |  |
| Intermediate | -0.16 -2.27 – 1.94 | 2.49 -0.88 – 5.86 | | 0.29 -1.25 – 1.83 | 1.13 -1.19 – 3.46 |
| Low-white collar | -1.76 -3.24 – -0.28 | 3.05 0.90 – 5.19 | | -0.82 -1.89 – 0.25 | 1.70 0.21 – 3.18 |
| Blue-collar | -0.10 -1.96 – 1.77 | 4.69 0.81 – 8.58 | | 0.45 -0.91 – 1.81 | 4.70 2.01 – 7.40 |
| **Residential education level (vs. high)** |  |  | |  |  |
| Medium-high | 0.59 -0.80 – 1.98 | -0.88 -3.07 – 1.30 | | 0.14 -0.85 – 1.14 | 0.19 -1.32 – 1.70 |
| Medium-low | 0.81 -0.69 – 2.31 | -2.91 -5.22 – -0.61 | | 0.89 -0.18 – 1.97 | 1.14 -0.44 – 2.73 |
| Low | 0.02 -1.55 – 1.60 | -1.73 -4.16 – 0.69 | | 1.13 0.01 – 2.26 | 2.28 0.59 – 3.97 |
| *Note.* CI, confidence interval. | | | | | |
