# Supplementary material for: Work economic sectors and cardiovascular risk factors: cross-sectional analysis based on the RECORD Study
Source: BMC Public Health. 2014 Jul 24;14:750. doi: 10.1186/1471-2458-14-750 (PMC4137071; doi:10.1186/1471-2458-14-750)
Supplement: Supplementary file 6 — Additional file 6: Associations between working in the secondary rather than in the tertiary sector and cardiovascular risk factors estimated from multilevel regression models among men and women. (DOCX 16 KB) [file 12889_2014_6938_MOESM6_ESM.docx]

| **Additional file 6** Associations between working in the secondary rather than in the tertiary sector and cardiovascular risk factors estimated from multilevel regression models among men and women* | | | | |
| --- | --- | --- | --- | --- |
|  | | **β (95% CI)** | | |
| **Cardiovascular risk factors** | | **Men** | | **Women** |
| BMI | | 0.14 -0.18 – 0.47 | | -0.50 -1.38 – 0.37 |
| Waist circumference | | 0.23 -0.72 – 1.18 | | -0.26 -2.30 – 1.79 |
| SBP | | 0.66 -0.68 – 2.01 | | 0.84 -2.40 – 4.08 |
| DBP | 0.77 -0.14 – 1.68 | | 0.14 -1.80 – 2.10 | |
| Pulse presure | -0.12 -0.99 – 0.76 | | 0.69 -1.49 – 2.88 | |
| Total cholesterol | 3.65 -0.08 – 7.39 | | | -1.48 -8.38 – 5.42 |
| Glycemia | -0.29 -1.58 – 1.00 | | | 0.36 -2.26 – 2.99 |
| HDL cholesterol | 0.84 -0.27 – 1.95 | | | -1.56 -7.70 – 4.58 |
| LDL cholesterol | 2.63 -0.63 – 5.91 | | | -1.76 -7.91 – 4.39 |
| Resting heart rate | -0.20 -1.13 – 0.73 | | | -0.64 -2.47 – 1.18 |
| *Model were adjusted for age, education, income, perceived financial strain, occupational status, and neighborhood level of education. Models for SBP, DBP, and pulse pressure were further adjusted for antihypertensive medication use.  *Note*. CI, confidence interval; BMI, body mass index; SBP, systolic blood pressure; DBP, diastolic blood pressure; HDL, high-density lipoprotein; LDL, low-density lipoprotein. | | | | |
